# A geospatial database of close-to-reality travel times to obstetric emergency care in 15 Nigerian conurbations

**DOI:** 10.1038/s41597-023-02651-9

**Published:** 2023-10-23

**Authors:** Peter M. Macharia, Kerry L. M. Wong, Tope Olubodun, Lenka Beňová, Charlotte Stanton, Narayanan Sundararajan, Yash Shah, Gautam Prasad, Mansi Kansal, Swapnil Vispute, Tomer Shekel, Uchenna Gwacham-Anisiobi, Olakunmi Ogunyemi, Jia Wang, Ibukun-Oluwa Omolade Abejirinde, Prestige Tatenda Makanga, Bosede B. Afolabi, Aduragbemi Banke-Thomas

**Affiliations:** 1 Department of Public Health, Institute of Tropical Medicine, Antwerp, Belgium; 2 Population & Health Impact Surveillance Group, Kenya Medical Research Institute-Wellcome Trust Research Programme, Nairobi, Kenya; 3 Centre for Health Informatics, Computing, and Statistics, Lancaster Medical School, Lancaster University, Lancaster, UK; 4 Faculty of Epidemiology and Population Health, London School of Hygiene and Tropical Medicine, London, UK; 5 Department of Community Medicine and Primary Care, Federal Medical Centre Abeokuta, Abeokuta, Ogun Nigeria; 6 Google LLC, California, USA; 7 Nuffield Department of Population Health, University of Oxford, Oxford, UK; 8 Lagos State Ministry of Health, Ikeja, Lagos, Nigeria; 9 School of Computing & Mathematical Sciences, University of Greenwich, London, UK; 10 Dalla Lana School of Public Health, University of Toronto, Toronto, Canada; 11 Women’s College Hospital Institute for Health System Solutions and Virtual Care, Toronto, Canada; 12 Surveying and Geomatics Department, Midlands State University Faculty of Science and Technology, Gweru, Midlands Zimbabwe; 13 Climate and Health Division, Centre for Sexual Health and HIV/AIDS Research, Harare, Zimbabwe; 14 Maternal and Reproductive Health Research Collective, Lagos, Nigeria; 15 Department of Obstetrics and Gynaecology, College of Medicine of the University of Lagos, Lagos, Nigeria; 16 School of Human Sciences, University of Greenwich, London, UK

**Keywords:** Health services, Health policy

## Abstract

Travel time estimation accounting for on-the-ground realities between the location where a need for emergency obstetric care (EmOC) arises and the health facility capable of providing EmOC is essential for improving pregnancy outcomes. Current understanding of travel time to care is inadequate in many urban areas of Africa, where short distances obscure long travel times and travel times can vary by time of day and road conditions. Here, we describe a database of travel times to comprehensive EmOC facilities in the 15 most populated extended urban areas of Nigeria. The travel times from cells of approximately 0.6 × 0.6 km to facilities were derived from Google Maps Platform’s internal Directions Application Programming Interface, which incorporates traffic considerations to provide closer-to-reality travel time estimates. Computations were done to the first, second and third nearest public or private facilities. Travel time for eight traffic scenarios (including peak and non-peak periods) and number of facilities within specific time thresholds were estimated. The database offers a plethora of opportunities for research and planning towards improving EmOC accessibility.

## Background & Summary

Evidence shows that timely access to emergency obstetric care (EmOC) provided by skilled health personnel, can reduce maternal deaths by 15–50% and intrapartum stillbirths by 45–75%^1^. EmOC is a set of nine clinical and surgical evidence-based interventions consisting of parenteral antibiotics, uterotonic drugs, parenteral anticonvulsants, manual removal of placenta, removal of retained products of conception, assisted vaginal delivery, neonatal resuscitation (altogether known as basic EmOC), blood transfusion and caesarean section (CS). The first seven interventions are collectively known as basic EmOC and the full set of nine are referred to as comprehensive EmOC. Globally, the met need for EmOC is only 45% with significant differences between low- and middle-income countries (LMICs) and high-income countries^2^. Basic EmOC is typically available in lower level health facilities such as primary health care centres and clinics while comprehensive EmOC tends to be provided only in public secondary and tertiary facilities and a number of private hospitals^3–5^.

However, before a pregnant woman with an obstetric emergency can access EmOC, she needs to travel to a health facility which can provide EmOC. This makes pertinent the questions of where the service is located (availability), the distance and time it takes to reach such services (accessibility). For those in urban areas, where multiple service locations are typical, authors have suggested that both availability and accessibility (referred to when combined as spatial accessibility) should be considered simultaneously^6–8^. As such, there is a clear need to understand spatial accessibility between the location where a need for obstetric emergency services arises and possible locations with comprehensive EmOC facilities^9,10^. Whether the woman accesses care through direct self-referral or institutional referral having previously been to another health facility^11^, the approach or method to estimate travel time in urban areas needs to consider complex factors such as traffic patterns and security concerns (e.g., during night-time^12^). Consequently, the approach adopted to estimate travel time has important implications for spatial metrics derived, and the subsequent policy decisions.

In literature and specifically in the context of LMICs, there are a range of approaches and methods that have been used to estimate spatial access to healthcare^8,13^. These primarily include modelled approaches and self-reported travel times^8,13^ varying from simple Euclidean distances^14,15^ to more complex cost distance algorithms^16^. However, the suitability of these methods is directly dependent on how well the models can be parametrised to accurately represent the dynamics of the journey between where the women is and the location of the EmOC facilities. This model parametrisation is achieved through data on the health-seeking behaviour and preferences of pregnant women and an accurate representation of road networks, land use, topography, and travel barriers.

Health-seeking data define parameters such as choices of pregnant women on whether to seek care, choices they make as to which facilities they trust to provide good-quality care, financial affordability, the means of transport and corresponding travel speeds for different road network classes and landcover categories^12,17^. However, there is a lack of such observational data on health-seeking behaviour to parametrise the models for the majority of the LMICs^18^. This challenge is more complicated in crowded, urban settings in Africa, where data availability is particularly limited. In urban areas, short distances may at times be obfuscated by long travel times, due to the variabilities and disruptions that might occur because of traffic congestion^19^, weather conditions, road accidents, police checkpoints, time of the day and day of the week when travelling occurs^9^. As a result, many models rely on average or plausibility-based speeds and modes of transport that are simplistic and underestimate travel times^20–23^. Thus, in a bid to improve access to EmOC services, policymakers often rely on inaccurate information or fail to rely on it because of the approximations^10,17,18^.

Beyond the complexities of estimating travel times in African urban settings, population growth justifies the focus on urban Africa. Two-thirds of the world’s population will live in urban areas by 2050 with a significant proportion of these additional 2.5 billion urban residents concentrating in Africa and tripling its urban population^24^. This is happening at a time when the use of health facilities for childbirth is near universal (>90%)^3^ in African urban conurbations (a city area containing many people, formed by various towns growing and merging (https://dictionary.cambridge.org/dictionary/english/conurbation)). Yet, emerging evidence shows that the odds of maternal death and stillbirth are significantly higher in urban areas compared to rural areas^25–27^. Consequently, spatial accessibility to EmOC in African urban settings has never been more crucial if we are to avert maternal and stillbirths and reach the 2030 Sustainable Development Goals (SDG)^28^ on maternal and newborn deaths.

Not only is Africa urbanising rapidly, but it also has the highest number of maternal deaths (69% of all 287,000 maternal deaths globally) and still births (49% of the 1.9 million stillbirths globally)^29–31^. The highest risk of maternal death and stillbirth is at the time of childbirth, from causes including severe bleeding, hypertensive disorders, obstructed labour, and sepsis^30,31^. Most of the 287,000 maternal and 1.9 million stillbirths^32^ can be prevented if women are able to access adequate care during pregnancy and give birth in facilities able to manage complications comprehensively^30,32^.

Therefore, understanding travel to EmOC in the African continent and in particular, urban and peri-urban areas is particularly important. There is an urgent need for estimates of travel time to EmOC facilities in African urban areas to account for on-the-ground realities^9^. The majority of previous and current travel time analyses^16,22,33–35^ have, however, not considered the time of the day (peak and non-peak hours), day of the week (weekend and weekday), weather and variations during transit to an EmOC facility. The current spatial accessibility models have mainly considered travel time to the nearest facility ignoring the alternatives such as the second and third closest facilities that a pregnant woman might prefer or choose^36^. Evidence shows women may bypass the nearest facility influenced by factors such as cost and quality of care^12,36,37^. Further, many of these analyses have only considered spatial accessibility to facilities in the public sector, ignoring the significant role played by the private health sector in expanding health service delivery in Africa^23^.

Given the need for updated and robust spatial access metrics, we developed and in this article describe a novel database capturing realistic estimates of travel time representing a significant improvement over earlier, comparable initiatives. The estimates capture travel time between cells (the size of each cell is approximately 0.6 square kilometres) and locations of EmOC facilities in the 15 most populated urban conurbations in Nigeria. These travel times were generated using Google Maps Platform’s internal Directions API (https://developers.google.com/maps/documentation/directions/overview) which uses machine Learning models that incorporates real-time traffic conditions along with historical traffic patterns and road network data to predict travel times^38^. The database focuses on Nigeria because it will have the third largest absolute increase in the size of the urban population, globally (highest increase in the urban population in Africa by 2050^24^) nearly doubling its urban dwellers between 2018 and 2050^24^. Additionally, of all African countries, Nigeria contributes the most to global maternal deaths (82,000) and stillbirths (182,307), globally^30,32^.

## Methods

### Overview

**Fig. 1:**
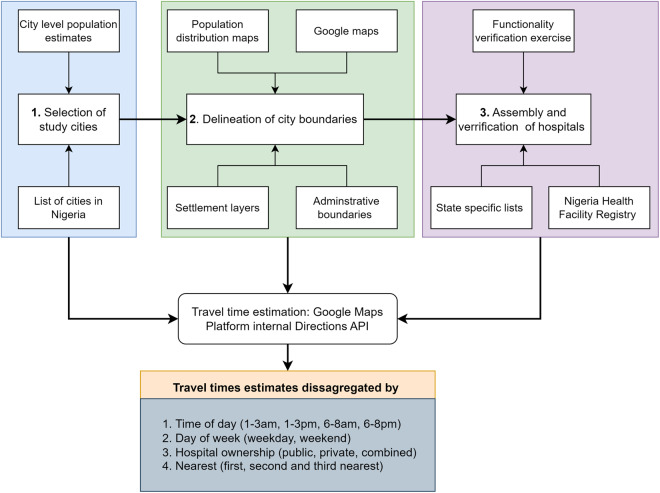
The methodological framework used to compute travel time in urban Nigeria. The framework includes selection of urban conurbations, delineation of urban conurbation boundaries, assembly and cleaning of health facilities, estimation of travel time and analysis based on various factors.

We followed a four-step process (Fig. 1) in developing this database incorporating closer-to-reality travel times in the 15 most populated Nigerian urban conurbations. It entailed i) selecting a subset of urban conurbations (the study areas) within Nigeria, ii) outlining the urban conurbation boundaries, iii) assembly, cleaning, and geocoding of hospitals capable of comprehensive EmOC services, and iv) applying the algorithm to estimate travel times between residence and service provider locations.

### Geographic scope and delineation of urban conurbation boundaries

The 15 urban conurbations included are Aba, Abuja, Benin City, Ibadan, Ilorin, Jos, Kaduna, Kano, Lagos, Maiduguri, Onitsha, Owerri, Port Harcourt, Uyo, and Warri. The inclusion criterion was based on a population threshold. That is, either the urban conurbation had an estimated population of least one million in 2022^24,39,40^ or its population is projected to reach the same threshold by 2030^24,39^ (Table 1), the SDG target year^28^. The justification for focusing on these conurbations is in recognition that they constitute areas where the effect of urbanisation and increasing population density are most acutely felt^41^. The maps of the selected urban areas are shown in the Supplementary file.

**Table 1:** Details of the urban conurbations included in the analysis.

ID	State	Urban conurbation	Number of Local Government Areas	Estimated population	
				2022	2030
1	Abia	Aba	6	1,150,116	1,527,000
2	Federal Capital Territory (FCT)	Abuja	8	3,652,029	5,119,000
3	Edo	Benin City	5	1,841,084	2, 451,000
4	Oyo	Ibadan	11	3,756,445	4, 956,000
5	Kwara	Ilorin	3	1,000,477	1, 314,000
6	Plateau	Jos	4	942,167	1,236,000
7	Kaduna	Kaduna	4	1,158,048	1, 499,000
8	Kano	Kano	16	4,219,209	5, 551,000
9	Lagos*	Lagos	20	15,387,639	20, 600,000
10	Borno	Maiduguri	2	822,337	1, 071,000
11	Anambra	Onitsha	4	1,552,630	2,138,000
12	Imo	Owerri	3	945,046	1,282,000
13	Rivers	Port Harcourt	9	3,324,694	4,595,000
14	Akwa-Ibom	Uyo	8	1,264,636	1,771,000
15	Delta	Warri	7	942,683	1,304,000

*The population of Lagos is disputed; some sources estimate the 2022 population to be over 26 million^54^.

The population dataset for each urban agglomeration was derived from the world city population^39^. The population data of the urban conurbations is based on the 2018 World Urbanisation Prospects by the United Nations Population Division^24^. In the prospectus, urban agglomeration incorporates the population in a city plus the population in the suburban areas lying outside but being adjacent to the city boundaries. The urban populations were extrapolated using a robust approach incorporating an average annual rate of change between urban-rural ratios that follows a logistic model and conforms to the worldwide observed pattern of urbanisation^24^.

**Fig. 2:**
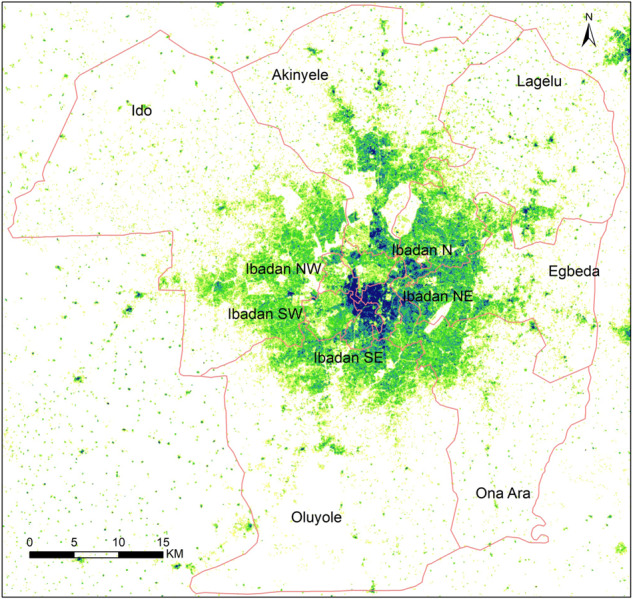
Ibadan urban area in Oyo state, Nigeria. Eleven Local Government Areas (LGAs) boundaries used to define Ibadan urban area spatially overlaid with population density ranging from low (yellow) to high density (dark blue).

There is no standard definition of urban conurbation boundaries, therefore, we used bespoke methods to delineate the geographic extents of each urban conurbation using existing layers of geospatial data. The ancillary data layers of population distribution showing counts of people^42^ and the Global Human Settlement Layer (GHSL) layer showing the degree of urbanisation^43^ were overlaid on Google Maps and Nigerian Local Government Area (LGA) boundaries^40^. The simultaneous spatial overlay of urban cells, agglomeration of people and built areas that intersected with an LGA formed the urban conurbation boundary. Provided part of the LGA was occupied by the spatial overlap of the proxies, the entire LGA was considered part of an urban area for policy relevance by the Federal Ministry of Health and state-level planners. Where necessary, in this study, several LGAs were merged to form the geographic extent of the urban conurbation. The defined geographical extent of the Ibadan urban conurbation in Oyo state is shown as an example in Fig. 2 while the maps of the other urban conurbation are shown in the Supplementary file.

### Geocoded database of health facilities capable of comprehensive EmOC services

Our main interest was to collate and geocode all health facilities from both public and non-public sectors capable of providing comprehensive EmOC for each of the 15 selected urban conurbations in Nigeria. We proxied and defined the availability of comprehensive EmOC services as any health facility that can offer CS services. The base list of health facilities was assembled from The Nigeria Health Facility Registry – NHFR (last updated in 2018)^44^. The NHFR contained information on the state, LGA, level of health facility, ownership, operation status, registration status, licence status, contact details and geographic location (latitude and longitude) of each facility. However, the NHFR did not have data on service availability including specific services such as CS, blood transfusion and operation hours (24 hours or not).

To determine if a health facility was capable of EmOC services (defined in this database by proxy of CS service availability since capacity for providing many other EmOC interventions is usually subsumed in capacity of facilities for CS^45^), we employed a range of approaches. For all urban conurbations, the main approach used was on-ground verification, in which research assistants visited the health facilities and administered short questionnaires to collect information on the availability of CS as well as hours of operation. In addition, when possible, additional verification of service availability in some facilities was done using the facility’s website or online health facility registries and by personal communication with physicians in the respective urban conurbations as well as phone calls to the respective health facilities. Further, specifically for Lagos, the Lagos State Health Facilities Monitoring and Accreditation Agency and the Lagos State Health Scheme databases which provided information on service availability and the state health insurance provider status, respectively, were used to complement our definition of service availability in health facilities.

The research assistants who conducted the on-ground verification of service availability were recruited from each of the 15 urban conurbations and included fifth- and sixth-year medical students, nurses, and medical doctors. The research assistants were trained on the administration of the questionnaire which contained questions on the alternative name of the facility, whether the health facility could provide a CS and its operating hours and the collection of the geolocation data of the health facility. Only tertiary, secondary facilities and privately owned primary facilities from the NHFR list were considered; public primary health centres and maternity homes were excluded. At each health facility, the research assistant acquired the necessary information from a senior medical staff e.g., a medical doctor or senior nurse. With the aid of a Geolocator mobile application, the geographic coordinates of each facility were collected while standing at the entrance of the health facility. A picture of the health facility showing the signpost was also taken.

After the removal of duplicates and harmonization, the final list of 2,021 functional health facilities contained attributes on facility code and name, location details (LGA name, latitude, and longitude) and ownership status. The list is publicly available^46^. Assembly was done using MS Excel (Microsoft, Redmond, USA).

### Computation of travel time

The travel time between approximately every 0.6 × 0.6 km cell (S2 level 14 cell (https://s2geometry.io/devguide/s2cell_hierarchy.html) (origin) - the resolution selected to balance between accuracy and computation needed for analysis - to locations of assembled EmOC facilities (destination) was computed using Google Maps Platform’s internal Directions API (https://developers.google.com/maps/documentation/directions/overview) for each urban conurbation. The S2 library defines a framework for decomposing the unit sphere into a hierarchy of cells where each cell is a quadrilateral bounded by four geodesics (https://s2geometry.io/devguide/s2cell_hierarchy.html). There are 30 levels of the S2 Cells, with the smallest at level 30. From each S2 cell, the API retrieved travel time from the cells to the destination by incorporating estimates of traffic to predict closer-to-reality travel time (https://developers.google.com/maps/documentation/directions/overview). This can help provide more realistic estimates given certain routes may be more affected by weather, accidents, or periodic traffic patterns at different time of the day. The equivalent external APIs providing such historical traffic data have been recently used to compute travel time to healthcare in LMIC settings^19,47^.

The API retrieved a driving travel time only, given that women in emergency conditions are likely to use a mode of transport that involved driving^48^ unless there is a part of the journey that is not motorable, where walking was assumed. Driving times were estimated from the centre of the S2 cell to the facility, given a particular day and time of the week. The retrieval call was done for every hour of the week: that is from Sunday at 12 am, 1 am, 2 am,…, and up to Saturday at 11 pm. The API calls were made in January 2023. This led to 168 travel times (7 days in a week × 24 hours per day) from each S2 cell to neighbouring facilities. For each S2 cell - facility pair, the median travel time was extracted by looking at the periodic distribution. That is, we extracted eight traffic scenarios (weekday/1–3 am, weekday/6–8 am, weekday/1–3 pm, weekday/6–8 pm, weekend/1–3 am, weekend/6–8 am, weekend/1–3 pm, weekend/6–8 pm). For example, for the “weekend/1–3 am” scenario, the median of the following six travel times was used: Sunday 1 am, 2 am, 3 am and Saturday 1 am, 2 am, and 3 am.

From the retrieved travel times, the following summaries were computed per S2 cell: i) travel times to the first, second, and third nearest facilities, recognising that pregnant women even in an emergency do not always travel to their nearest facility^12^ and ii) the count of the number of health facilities within 15, 30, and 60 minutes of each S2 cell, keeping in mind that poor pregnancy outcomes like stillbirths and maternal deaths have been reported after self-referral and institutional referral respectively at these lower travel time benchmarks in urban African conurbations^26,27^. The nearest facilities were defined as the ones that required the shortest travel time to get to. These estimates were disaggregated by facility ownership (public, private and a combination of both).

## Data Records

The database of public and private EmOC facilities^46^ and that of travel time^49^ resulting from the described process have been made publicly and freely available through the Figshare repositories^46,49^. The travel time database includes data from 15 urban conurbations in Nigeria. Each data record represents a travel time estimate from 0.6 km square cell to a specific comprehensive EmOC facility (private, public or either) at a particular time of the day and week. The database has 16 descriptive variables summarised in Table 2. The World Geodetic System 1984 (WGS84) coordinate system was adopted for the presentation of any geographical coordinates, at six decimal places.

**Table 2:** Description of variables in the travel time database.

ID	Variable	Description and options
1	S2cellid	The ID of the 0.6 km^2^ cell (unit of analysis from which travel time was computed)
2	Center_lat_lng	Location attribute -the centre coordinates (latitude and longitude) of the cell
3	Facility_type	Three options that specify whether the destination facility from the cell is public, private or both sectors
4	Departure_time	Eight options showing combination of time of day, and day of week when journey is made: weekday/1–3 am, weekday/6–8 am, weekday/1–3 pm, weekday/6–8 pm, weekend/1–3 am, weekend/6–8 am, weekend/1–3 pm, and weekend/6–8 pm
5	n_within_15	Number of facilities within 15 minutes of the reference cell (cell ID and its coordinates) for each category of facility ownership
6	n_within_30	Number of facilities within 30 minutes of the reference cell (cell ID and its coordinates) for each category of facility ownership
7	n_within_60	Number of facilities within 60 minutes of the reference cell (cell ID and its coordinates) for each category of facility ownership
8	Min_travel_time_1_minutes	Travel time in minutes to the 1^st^, 2^nd^ and the 3^rd^ nearest facility, respectively to the reference cell.
9	Min_travel_time_2_minutes	
10	Min_travel_time_3_minutes	
11	Nearest_facility_1	Unique code of the 1^st^, 2^nd^, and 3^rd^ nearest facility, respectively. The codes can be used to link with main facility database^16^
12	nearest_facility_2	
13	nearest_facility_3	
14	nearest_facility_1_coordinates	Geographic coordinates of the 1^st,^ 2^nd^, and 3^rd^ nearest facility to the reference cell.
15	nearest_facility_2_coordinates	
16	nearest_facility_3_coordinates	

## Technical Validation

We validated the functionality and capacity of the health facility to perform comprehensive EmOC services as elaborated under the assembly of a geocoded database of health facilities capable of EmOC services^46^. To validate the extracted travel time from the internal API, we randomly picked locations in each of the conurbation and manually extracted corresponding travel (particular day of the week and time of the day) from Google Maps, using the typical travel time on the front end of Google Maps, as we have done previously^50^.

## Usage Notes

Closer-to-reality estimates of travel time, using the Google Maps external Directions API or manually extracting travel time from Google Maps, have been used to guide stakeholders to comprehensively explore geographical inequities in healthcare access including EmOC within urban settings and inform policy and targeted planning^47,50^. Such results when presented in a dynamic way have been deemed particularly valuable by policymakers^51^. The provided travel time data sets can be summarized by aggregating at any subnational region of choice for example at the LGA, ward or urban conurbation-level using any statistical or any geospatial mapping software via the Zonal statistics functionalities.

The geographic coordinates can be used to link the database with existing population distribution data^52^, compute spatial coverage estimates and other socio-demographic datasets such as the relative wealth index^53^. While the dataset does not refer to any residential addresses, it can be spatially linked with external household surveys to derive their travel times. The database, however, contains driving as the only mode of transport for a very specific selection of health facilities and cannot be generalised.

Eight traffic scenarios were considered during data generation. However, for data summary purposes, in this data descriptor, we refer to peak as Friday at 6 p.m. (the departure day and time with the longest travel time across all the generated travel time scenarios) and non-peak as Sunday at 4 a.m. (the departure day and time with the shortest travel time across all the generated travel time scenarios).

The estimates are available at 0.6 by 0.6 km grids, a spatial resolution that balanced our computational power and efficiency and allows more granular understanding of accessibility. This is an improvement in global and regional studies that have used courser grids^21,22,34^.

### Supplementary information

Supplementary file 1

## Data Availability

No custom code was developed since the extraction of travel time was done through the Google Maps internal Directions API (https://developers.google.com/maps/documentation/directions/overview).
