# Supplementary material for: A geospatial database of close-to-reality travel times to obstetric emergency care in 15 Nigerian conurbations
Source: Sci Data. 2023 Oct 23;10:736. doi: 10.1038/s41597-023-02651-9 (PMC10593805; doi:10.1038/s41597-023-02651-9)

Supplementary file

Macharia et al. A geospatial database of close-to-reality travel times to obstetric emergency care in 15 Nigerian conurbations. Scientific Data. (2023).

Table of Contents

Figure S1. The local government area boundaries that defined the urban spatial extents of Aba city in Abia state, Nigeria, with spatial representation of its population distribution. 2

Figure S2. The local government area boundaries that defined the urban spatial extents of Abuja the Federal Capital Territory, Nigeria, with spatial representation of its population distribution. 3

Figure S3. The local government area boundaries that defined the urban spatial extents of Benin City in Edo state, Nigeria, with spatial representation of its population distribution. 4

Figure S4. The local government area boundaries that defined the urban spatial extents of Ibadan in Oyo state, Nigeria, with spatial representation of its population distribution. 5

Figure S5. The local government area boundaries that defined the urban spatial extents of Ilorin in Kwara state, Nigeria, with spatial representation of its population distribution. 6

Figure S6. The local government area boundaries that defined the urban spatial extents of Jos in Plateau state, Nigeria, with spatial representation of its population distribution. 7

Figure S7. The local government area boundaries that defined the urban spatial extents of Kaduna in Kaduna state, Nigeria, with spatial representation of its population distribution. 8

Figure S8. The local government area boundaries that defined the urban spatial extents of Kano in Kano state, Nigeria, with spatial representation of its population distribution. 9

Figure S9. The local government area boundaries that defined the urban spatial extents of Legos in Lagos state, Nigeria, with spatial representation of its population distribution. 10

Figure S10. The local government area boundaries that defined the urban spatial extents of Maiduguri in Borno state, Nigeria, with spatial representation of its population distribution. 11

Figure S11. The local government area boundaries that defined the urban spatial extents of Onitsha in Anambra state, Nigeria, with spatial representation of its population distribution. 12

Figure S12. The local government area boundaries that defined the urban spatial extents of Owerri in Imo state, Nigeria, with spatial representation of its population distribution. 13

Figure S13. The local government area boundaries that defined the urban spatial extents of Port Harcourt in Rivers state, Nigeria, with spatial representation of its population distribution. 14

Figure S14. The local government area boundaries that defined the urban spatial extents of Uyo in Akwa-Ibom state, Nigeria, with spatial representation of its population distribution. 15

Figure S15. The local government area boundaries that defined the urban spatial extents of Warri in Delta state, Nigeria, with spatial representation of its population distribution. 16

Figure S1. The local government area boundaries that defined the urban spatial extents of Aba city in Abia state, Nigeria, with spatial representation of its population distribution.

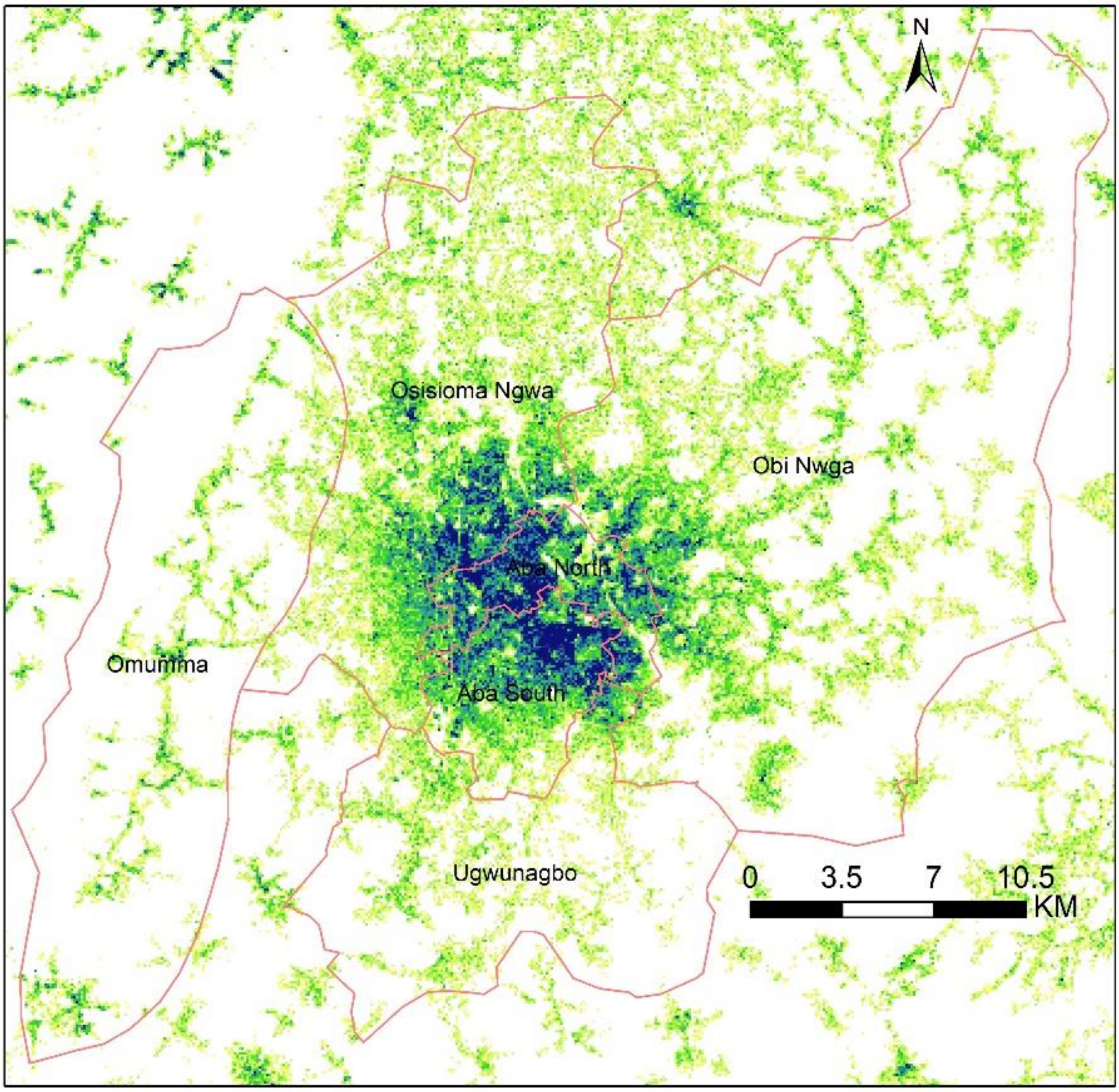

Figure S2. The local government area boundaries that defined the urban spatial extents of Abuja the Federal Capital Territory, Nigeria, with spatial representation of its population distribution.

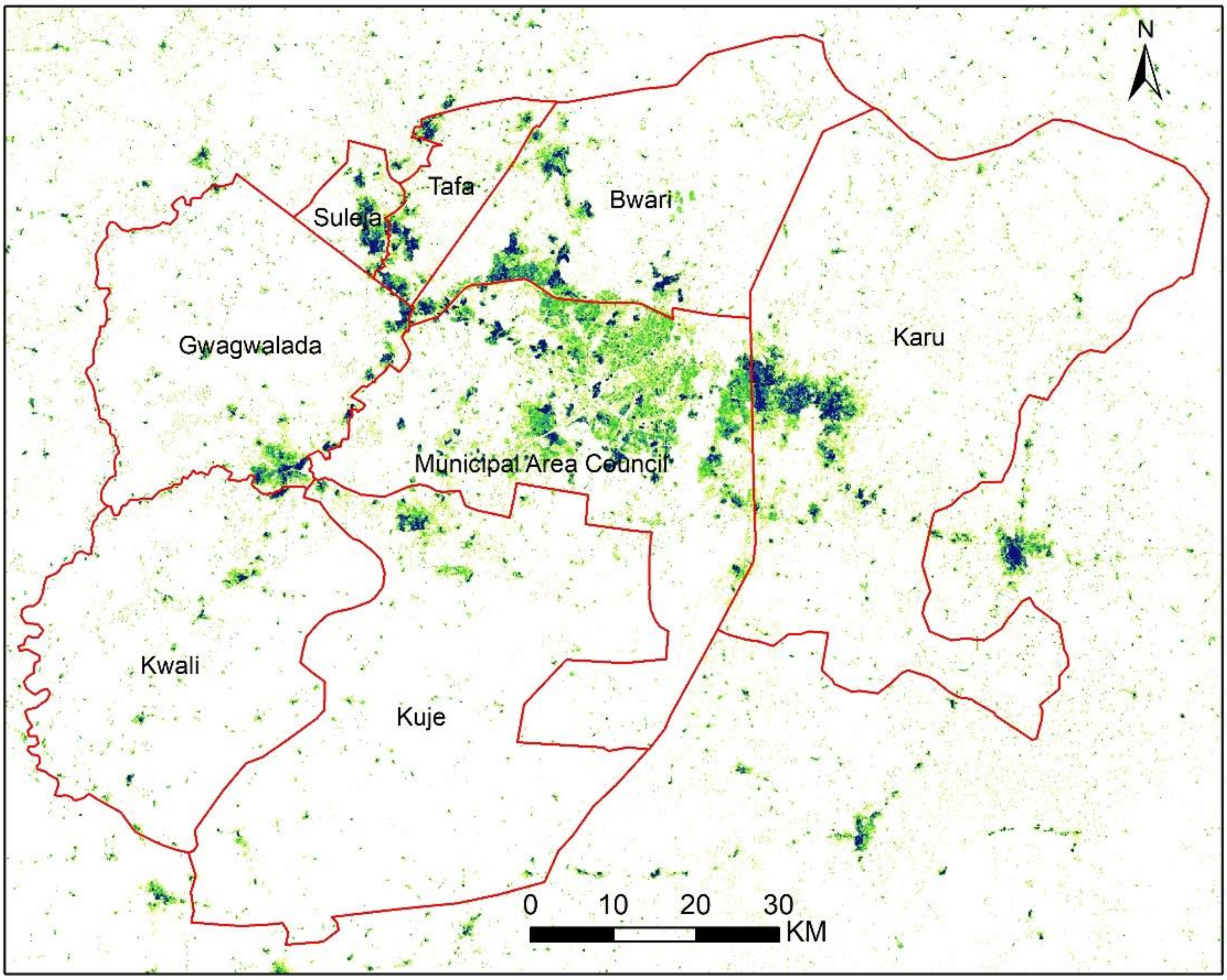

Figure S3. The local government area boundaries that defined the urban spatial extents of Benin City in Edo state, Nigeria, with spatial representation of its population distribution.

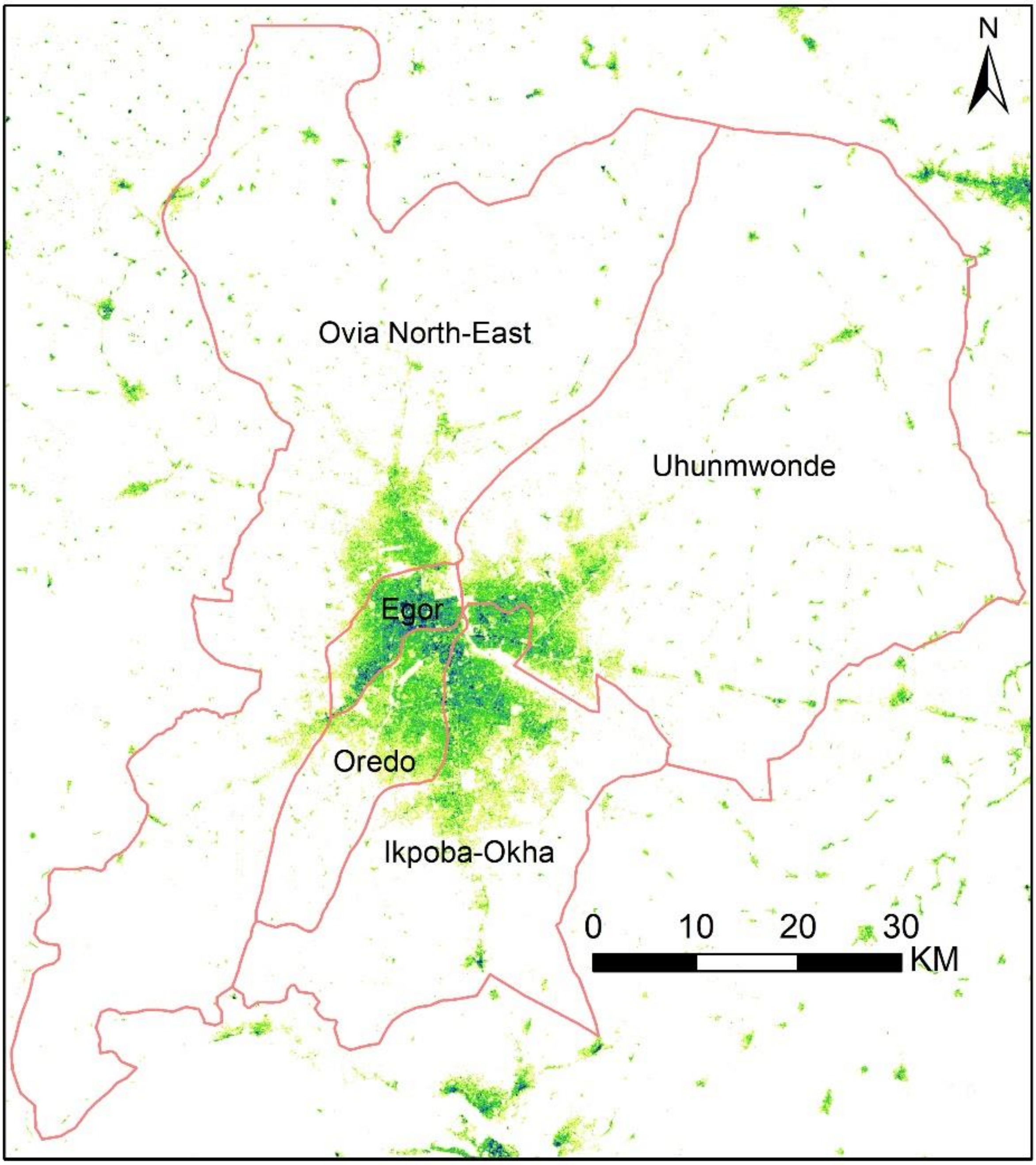

Figure S4. The local government area boundaries that defined the urban spatial extents of Ibadan in Oyo state, Nigeria, with spatial representation of its population distribution.

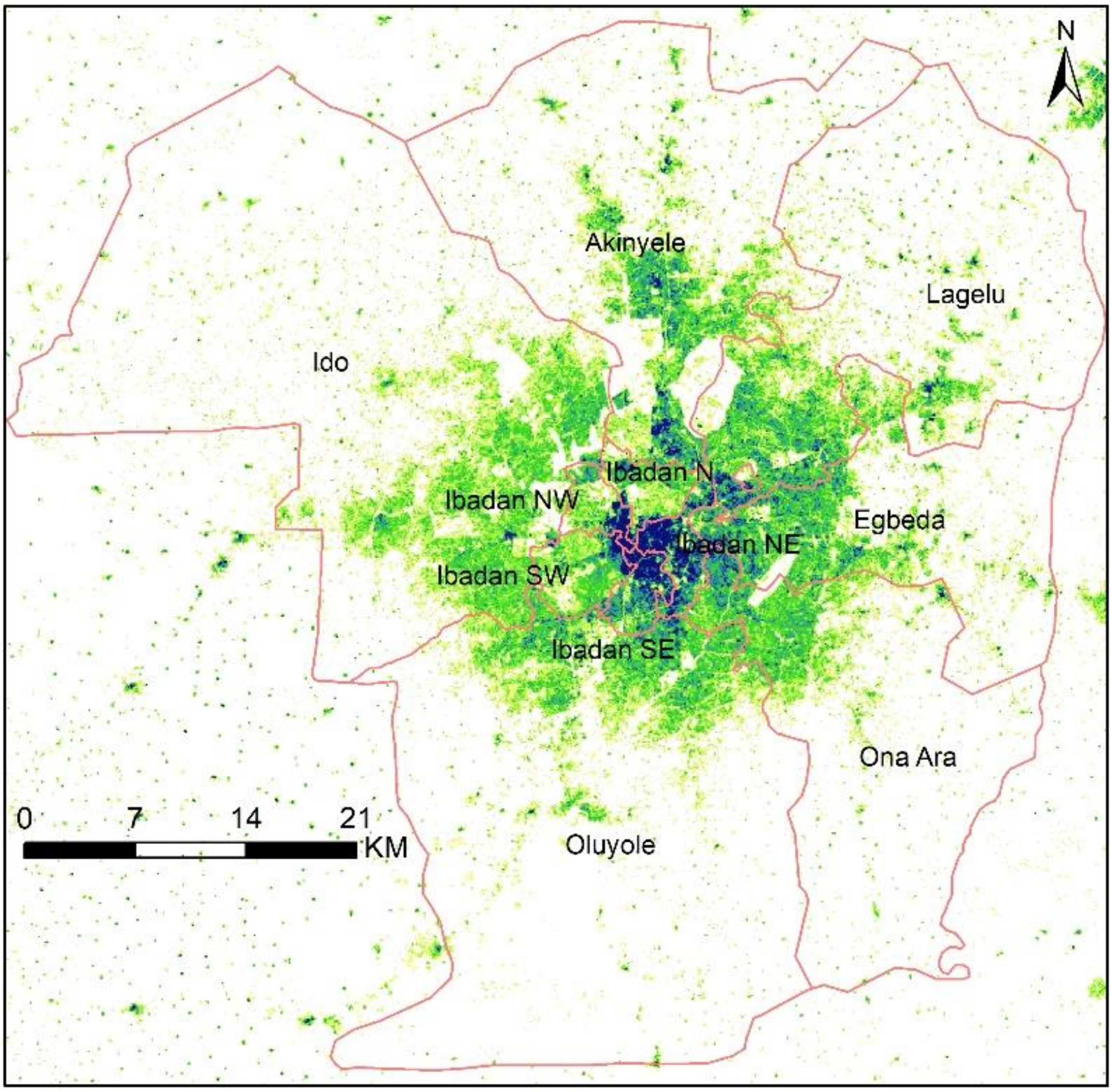

Figure S5. The local government area boundaries that defined the urban spatial extents of Ilorin in Kwara state, Nigeria, with spatial representation of its population distribution.

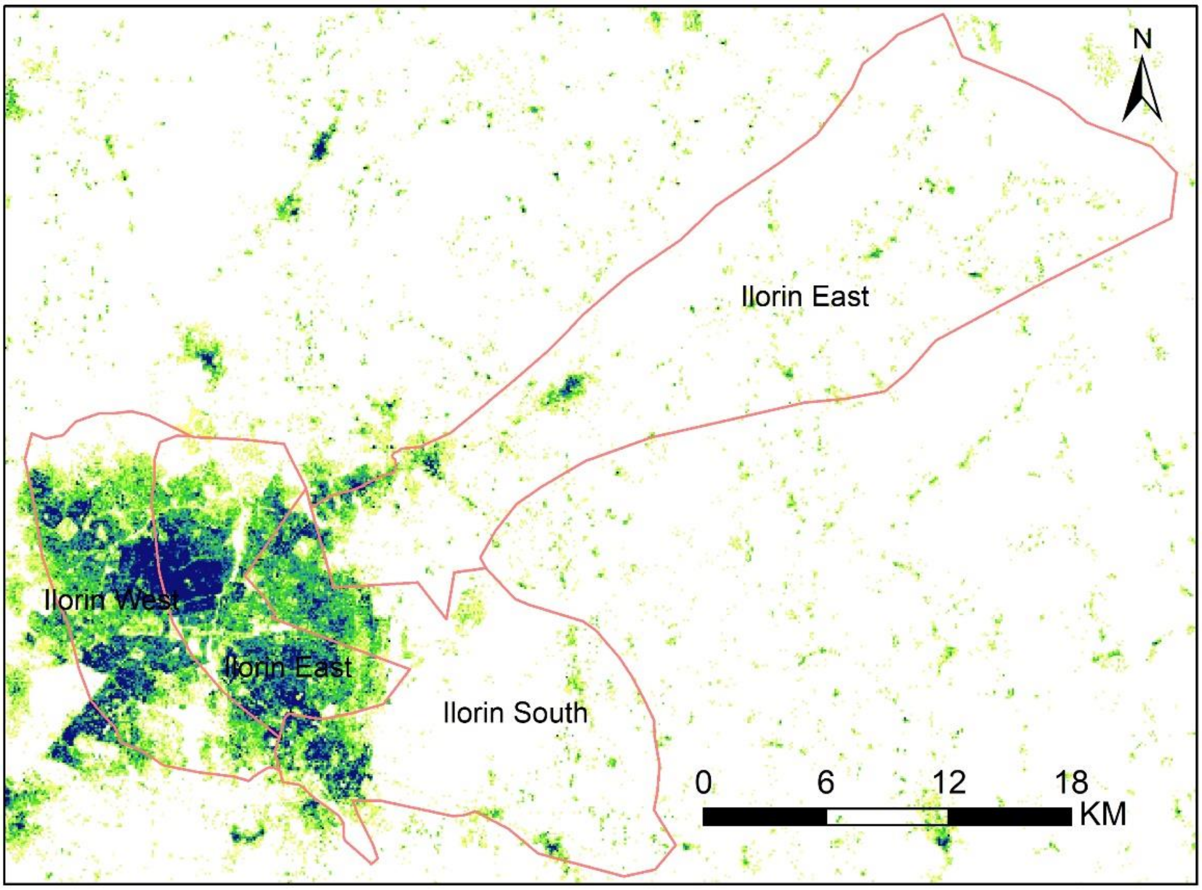

Figure S6. The local government area boundaries that defined the urban spatial extents of Jos in Plateau state, Nigeria, with spatial representation of its population distribution.

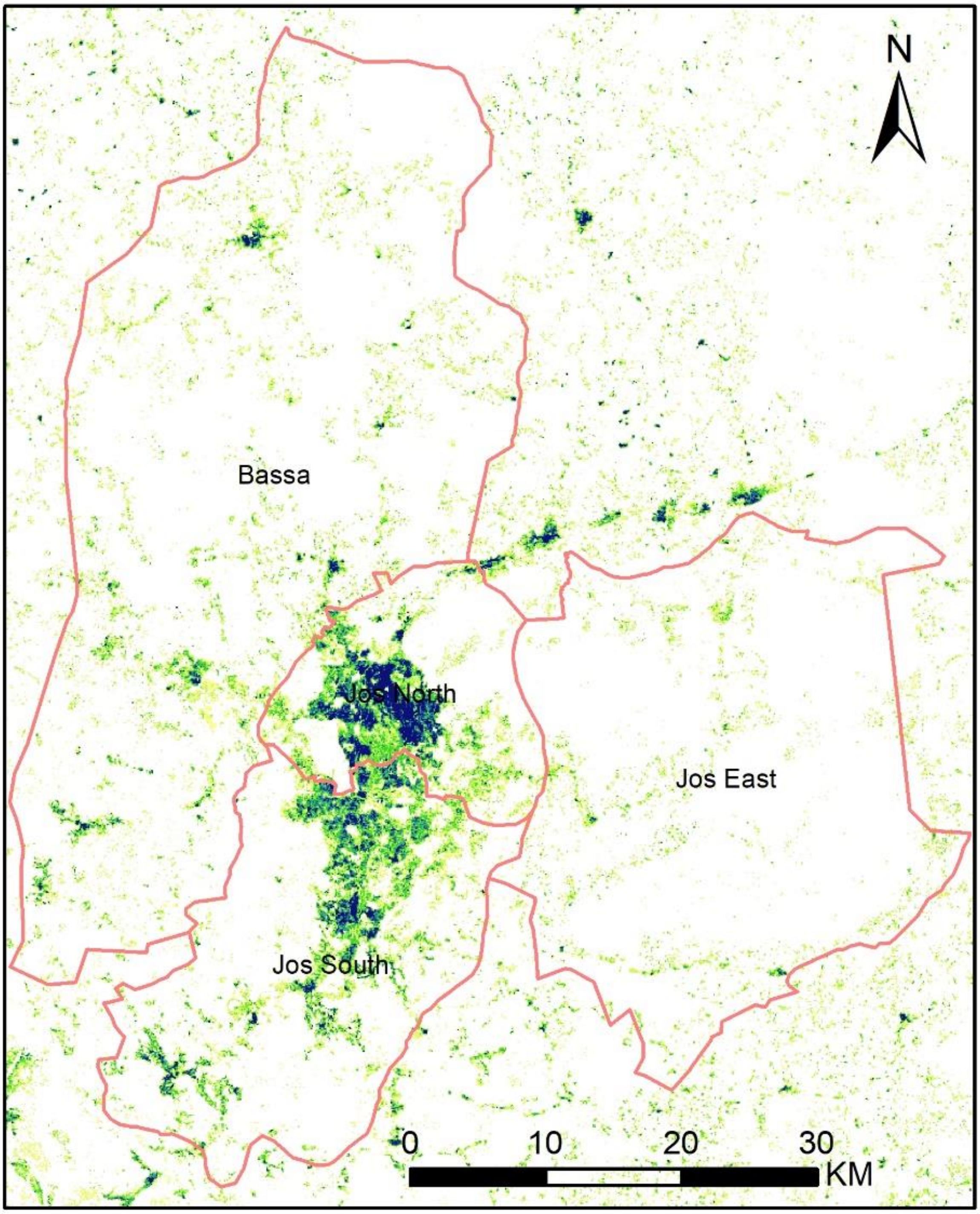

Figure S7. The local government area boundaries that defined the urban spatial extents of Kaduna in Kaduna state, Nigeria, with spatial representation of its population distribution.

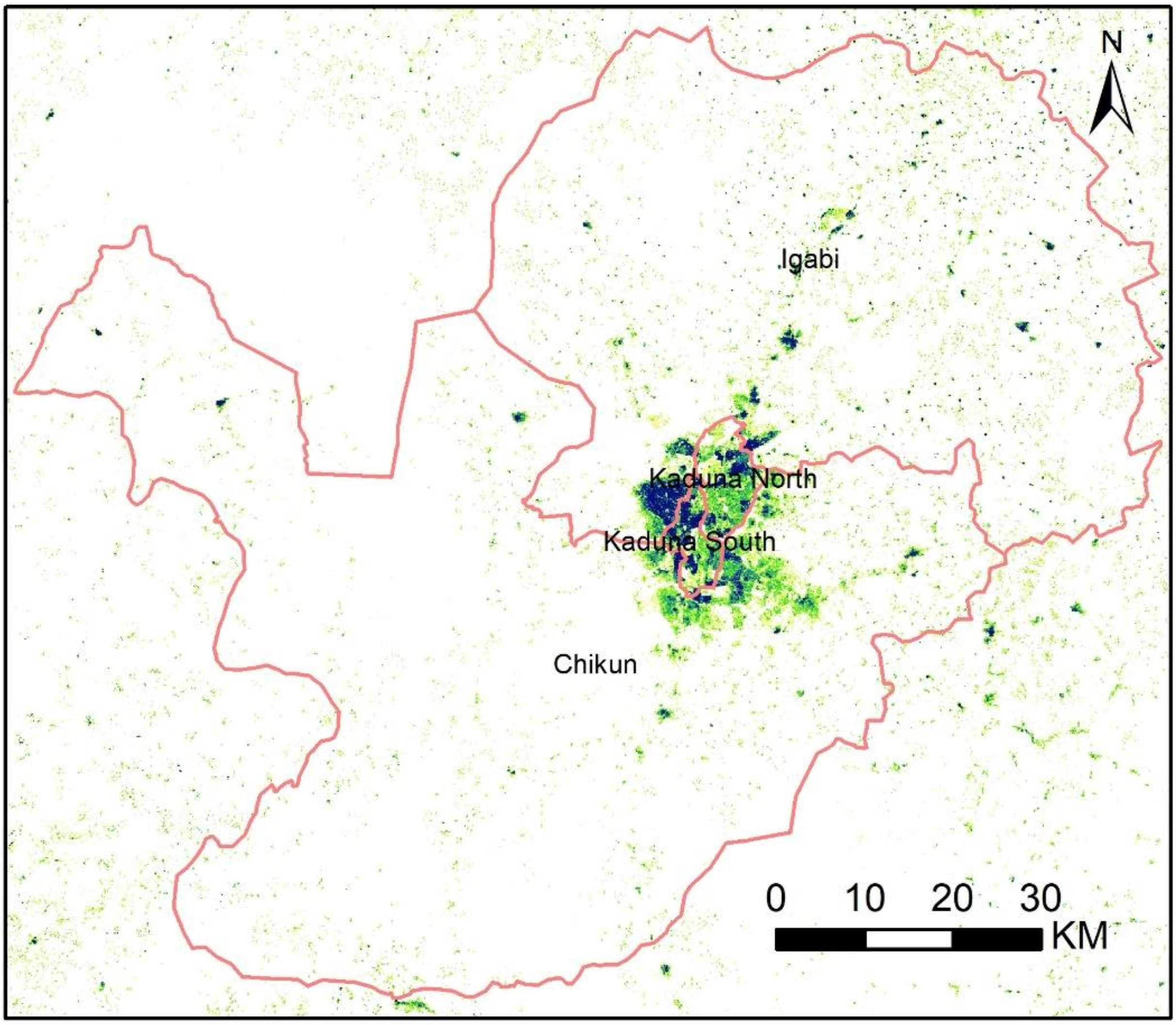

Figure S8. The local government area boundaries that defined the urban spatial extents of Kano in Kano state, Nigeria, with spatial representation of its population distribution.

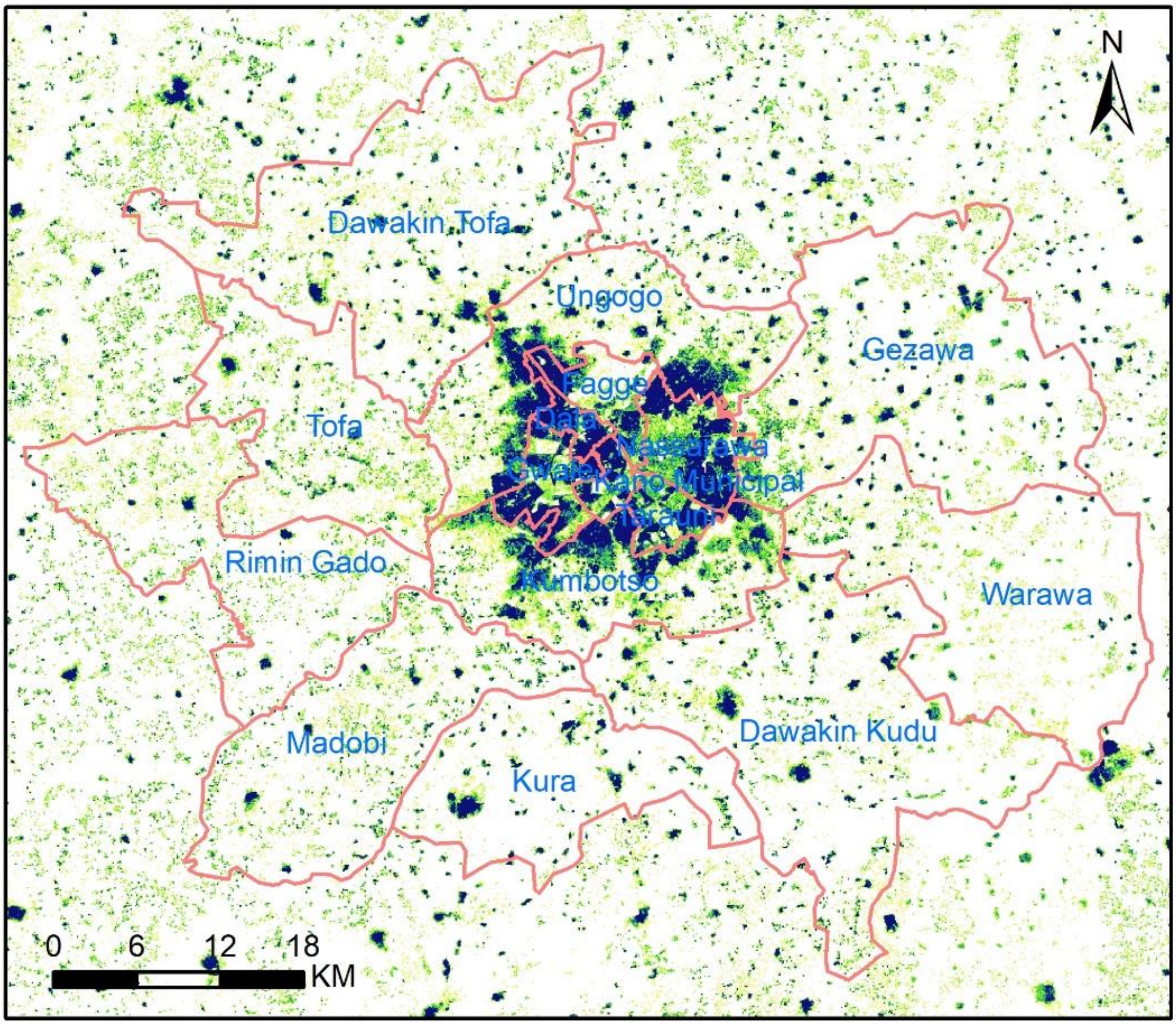

Figure S9. The local government area boundaries that defined the urban spatial extents of Legos in Lagos state, Nigeria, with spatial representation of its population distribution.

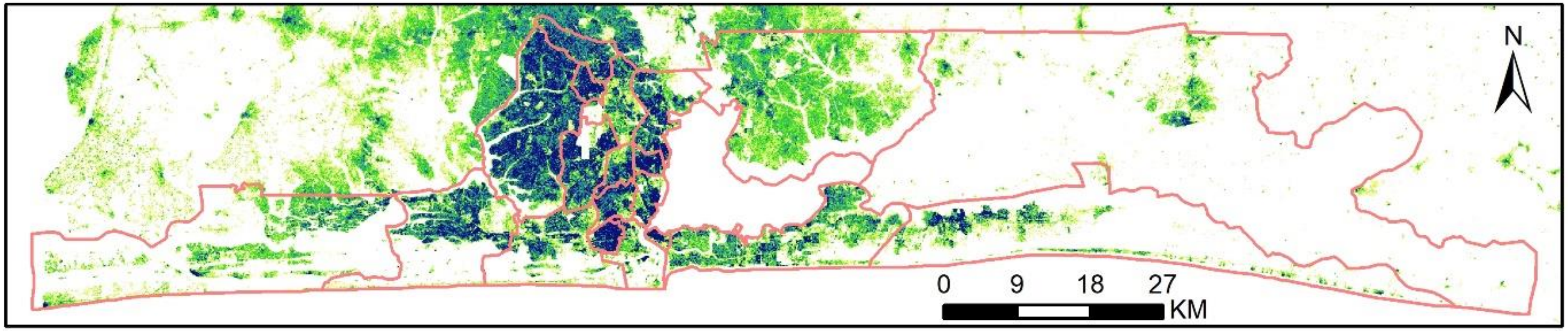

Figure S10. The local government area boundaries that defined the urban spatial extents of Maiduguri in Borno state, Nigeria, with spatial representation of its population distribution.

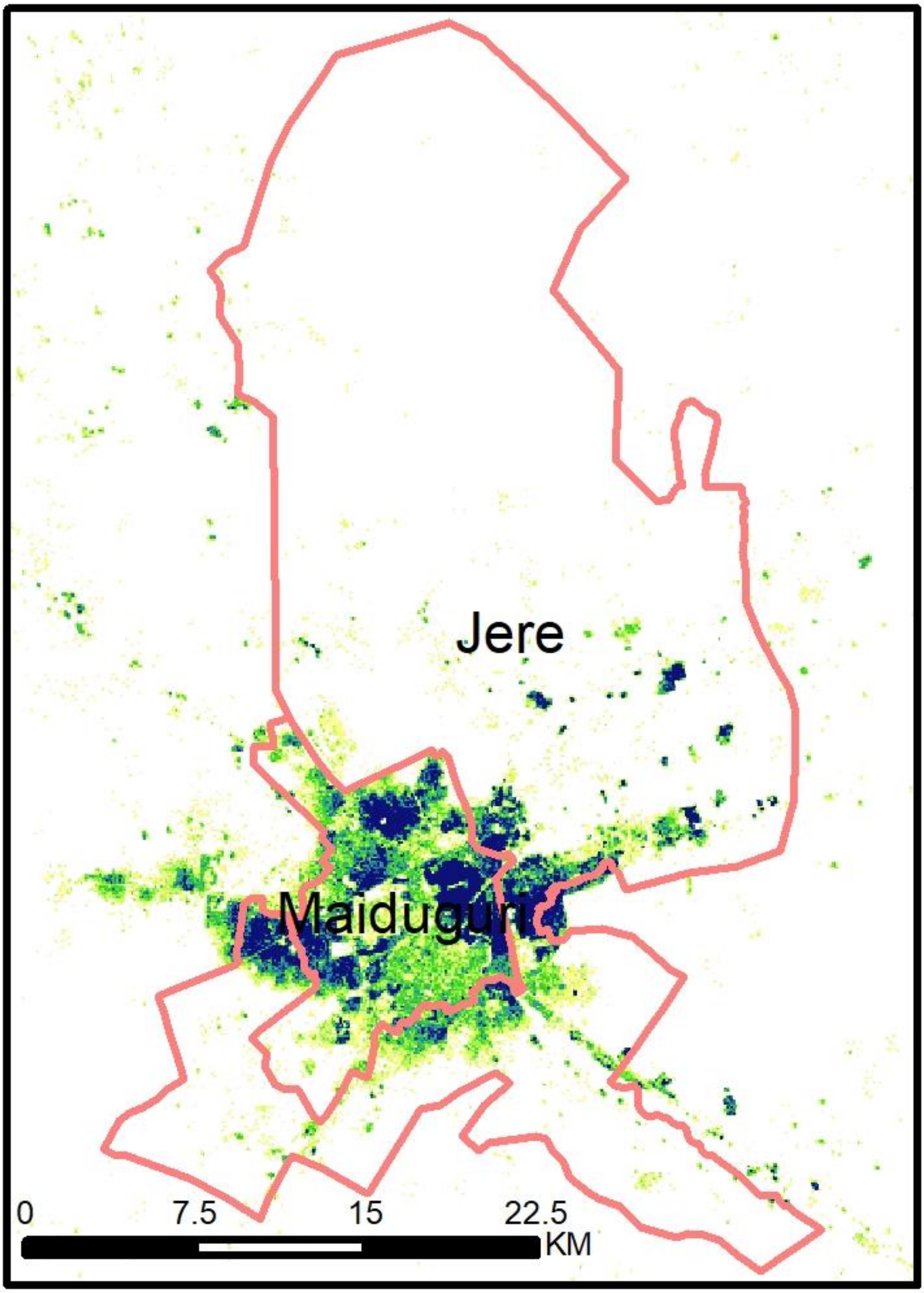

Figure S11. The local government area boundaries that defined the urban spatial extents of Onitsha in Anambra state, Nigeria, with spatial representation of its population distribution.

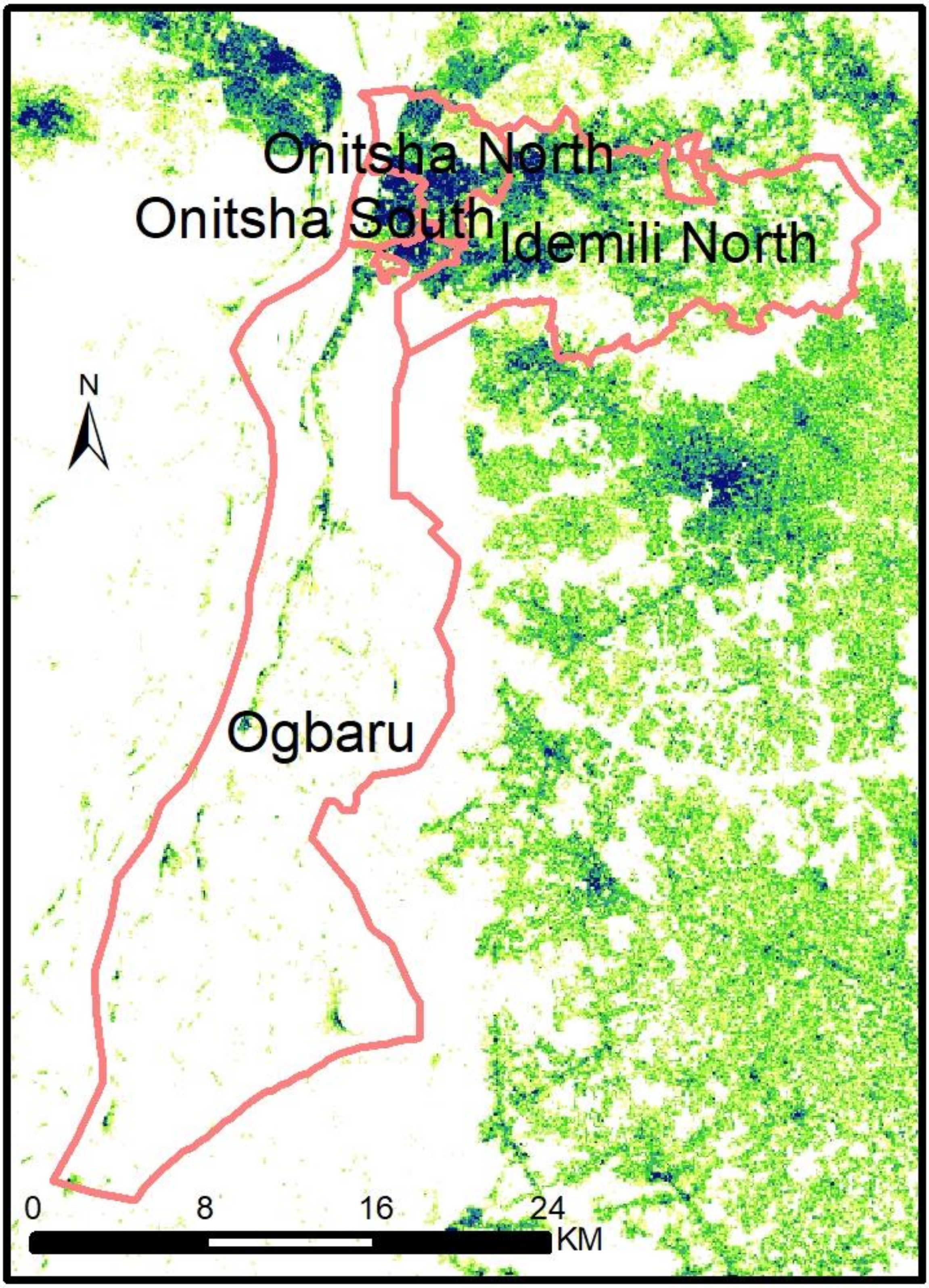

Figure S12. The local government area boundaries that defined the urban spatial extents of Owerri in Imo state, Nigeria, with spatial representation of its population distribution.

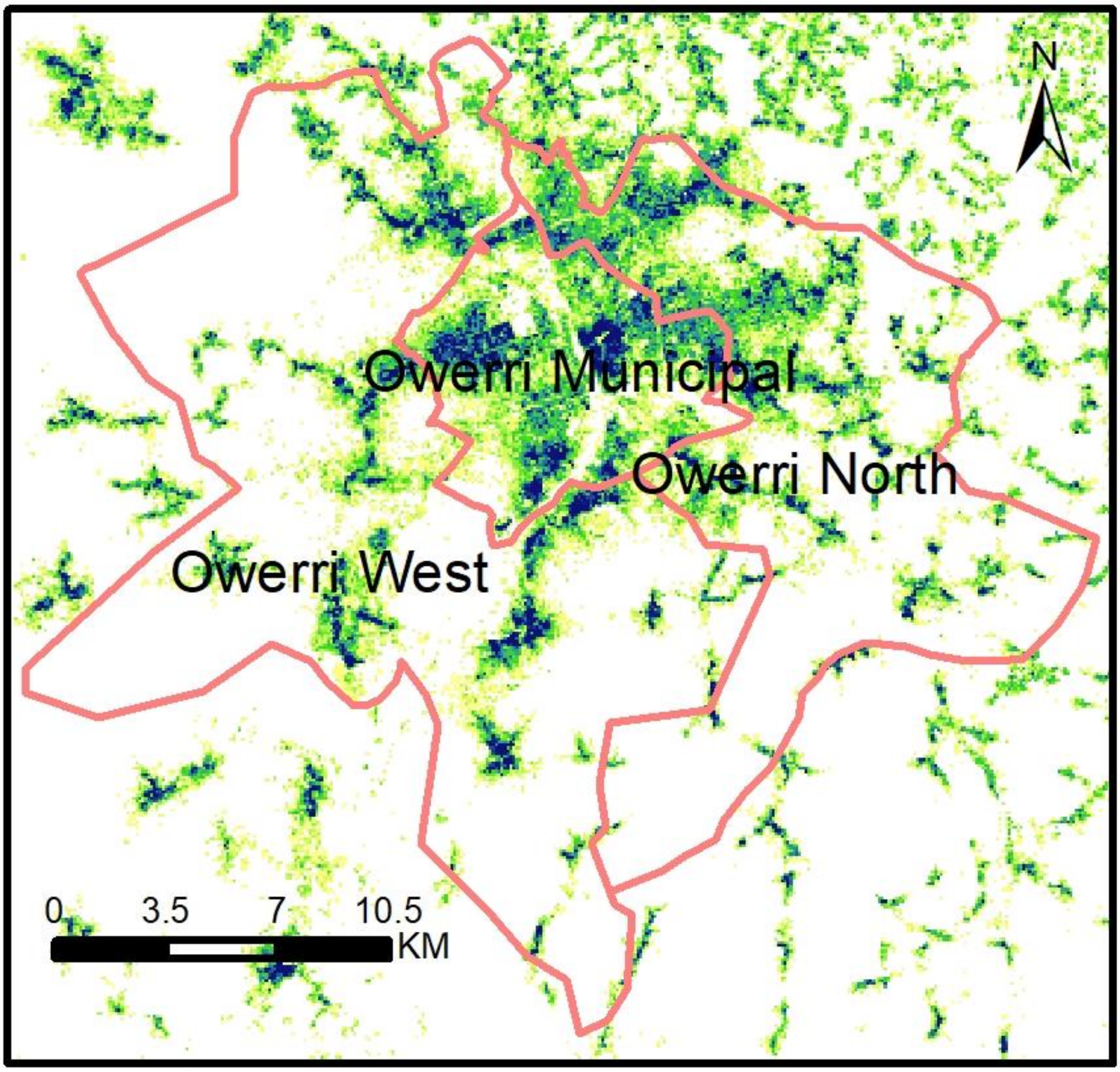

Figure S13. The local government area boundaries that defined the urban spatial extents of Port Harcourt in Rivers state, Nigeria, with spatial representation of its population distribution.

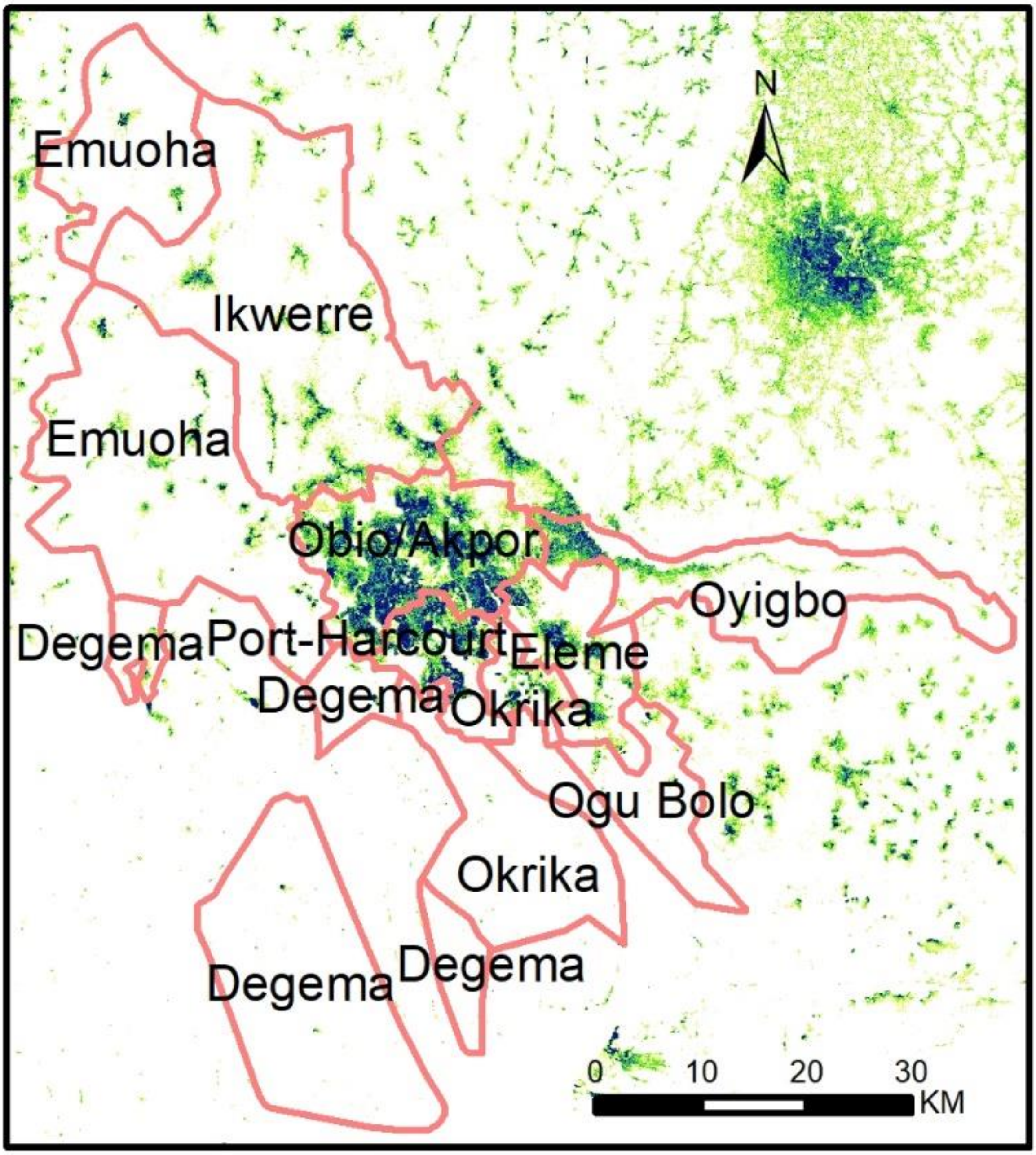

Figure S14. The local government area boundaries that defined the urban spatial extents of Uyo in Akwa-Ibom state, Nigeria, with spatial representation of its population distribution.

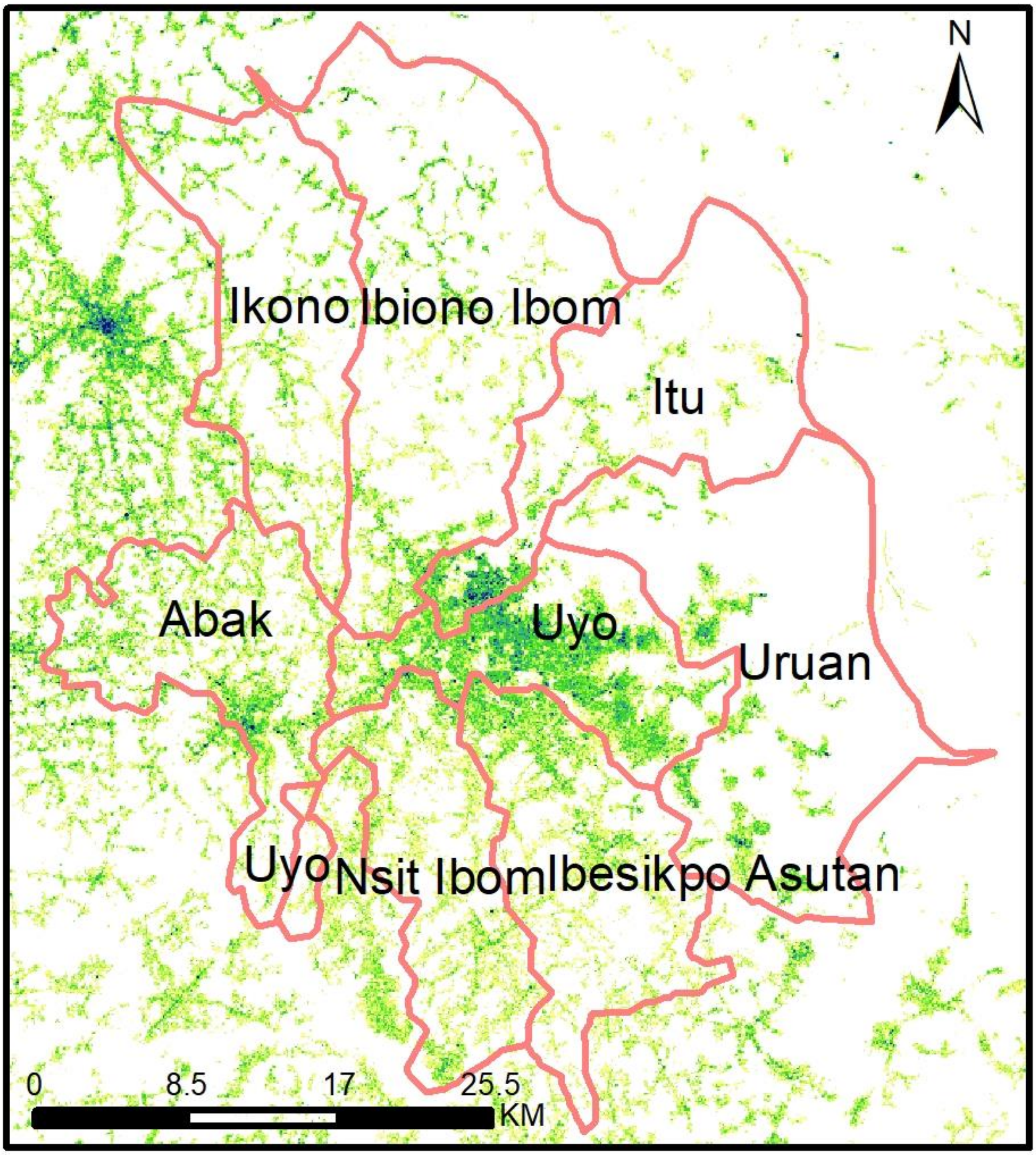

Figure S15. The local government area boundaries that defined the urban spatial extents of Warri in Delta state, Nigeria, with spatial representation of its population distribution.

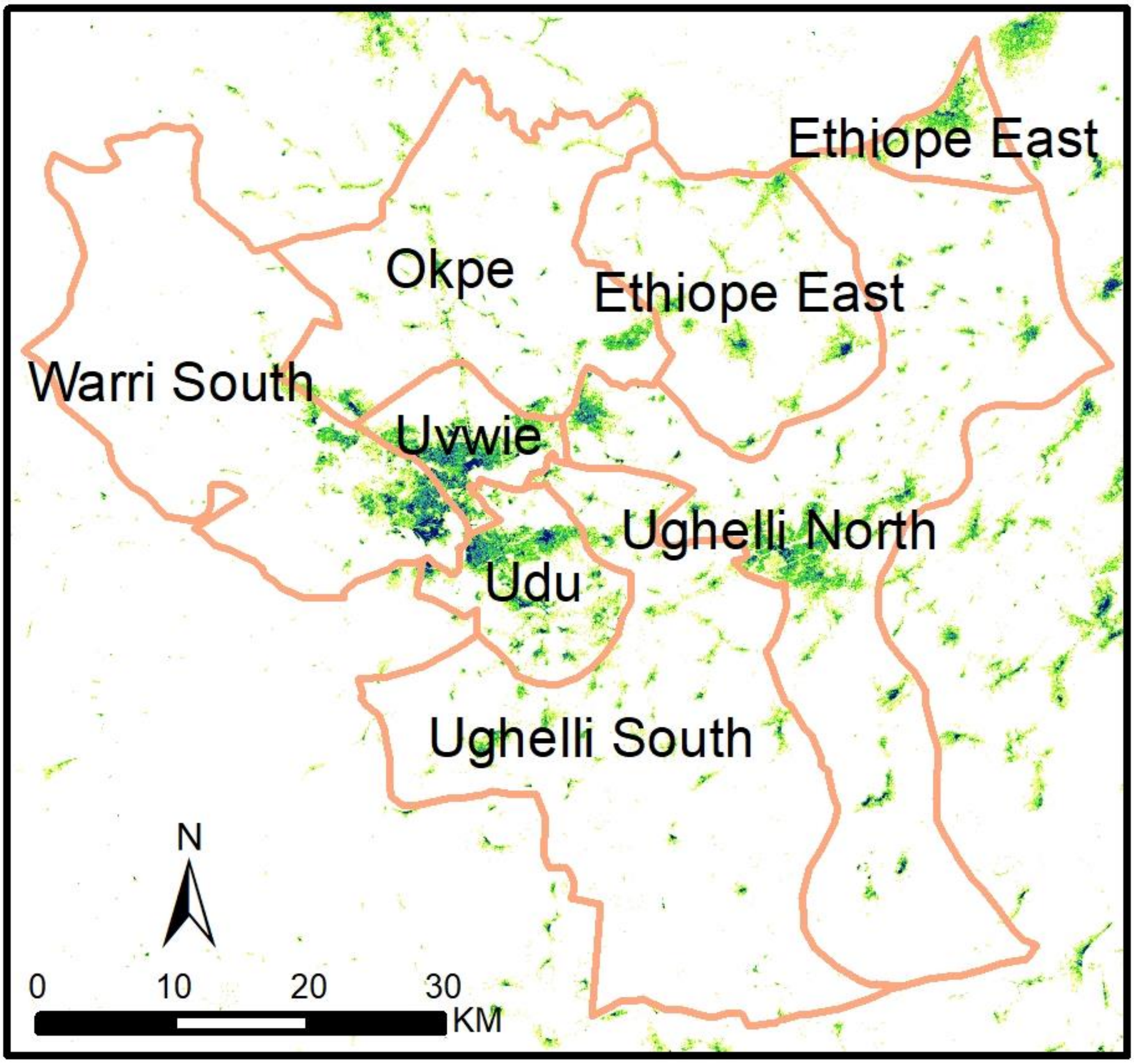

Supplement: Supplementary file 1 [file 41597_2023_2651_MOESM1_ESM.pdf]
